# The Importance of Entomo-Virological Investigation of Yellow Fever Virus to Strengthen Surveillance in Brazil

**DOI:** 10.3390/tropicalmed8060329

**Published:** 2023-06-20

**Authors:** Ana Cecília Ribeiro Cruz, Leonardo Henrique Almeida Hernández, Carine Fortes Aragão, Thito Yan Bezerra da Paz, Sandro Patroca da Silva, Fábio Silva da Silva, Ana Alice de Aquino, Glennda Juscely Galvão Pereira Cereja, Bruna Lais Sena do Nascimento, José Wilson Rosa Junior, Carmeci Natalina Elias, Cristiano Gomes Nogueira, Daniel Garkauskas Ramos, Vagner Fonseca, Marta Giovanetti, Luiz Carlos Junior Alcantara, Bruno Tardelli Diniz Nunes, Pedro F. da Costa Vasconcelos, Livia Carício Martins, Joaquim Pinto Nunes-Neto

**Affiliations:** 1 Department of Arbovirology and Hemorrhagic Fevers, Evandro Chagas Institute, Health and Environment Surveillance Secretariat, Ministry of Health, Ananindeua 67030-000, PA, Brazil; 2 Center for Biological and Health Sciences, Pará State University, Belém 66087-670, PA, Brazil; 3 Institute of Biological Sciences, Federal University of Pará, Belém 66075-110, PA, Brazil; 4 Goiás Public Health Laboratory, Goiânia 74853-120, GO, Brazil; 5 Health and Environment Surveillance Secretariat, Ministry of Health, Brasília 70723-040, DF, Brazil; 6 Public Health Emergency Department, Pan American Health Organization, World Health Organization, Brasília 70800-400, DF, Brazil; 7 René Rachou Institute, Oswaldo Cruz Foundation, Belo Horizonte 30190-002, MG, Brazil

**Keywords:** yellow fever virus, vectors, *Aedes albopictus*, outbreak, entomo-virological surveillance

## Abstract

The largest outbreak of sylvatic yellow fever virus (YFV) in eight decades was recorded in Brazil between 2016–2018. Besides human and NHP surveillance, the entomo-virological approach is considered as a complementary tool. For this study, a total of 2904 mosquitoes of the *Aedes*, *Haemagogus* and *Sabethes* genera were collected from six Brazilian states (Bahia, Goiás, Mato Grosso, Minas Gerais, Pará, and Tocantins) and grouped into 246 pools, which were tested for YFV using RT-qPCR. We detected 20 positive pools from Minas Gerais, 5 from Goiás, and 1 from Bahia, including 12 of *Hg. janthinomys* and 5 of *Ae. albopictus*. This is the first description of natural YFV infection in this species and warns of the likelihood of urban YFV re-emergence with *Ae. albopictus* as a potential bridge vector. Three YFV sequences from *Hg. janthinomys* from Goiás and one from Minas Gerais, as well as one from *Ae. albopictus* from Minas Gerais were clustered within the 2016–2018 outbreak clade, indicating YFV spread from Midwest and its infection in a main and likely novel bridging vector species. Entomo-virological surveillance is critical for YFV monitoring in Brazil, which could highlight the need to strengthen YFV surveillance, vaccination coverage, and vector control measures.

## 1. Introduction

The yellow fever virus (YFV), *Orthoflavivirus flavi*, the prototype virus of the *Orthoflavivirus* genus and *Flaviviridae* family [1], is an endemic arbovirus in tropical and subtropical countries. In Brazil, YFV is historically maintained through urban and sylvatic transmission cycles. The urban cycle, which had not been recorded in Brazil since 1942 and in the Americas (Trinidad and Tobago) since 1954, involves transmission between *Aedes aegypti* mosquitoes, the main vector, and humans. In the sylvatic cycle, *Haemagogus* and *Sabethes* genera mosquitoes act as vectors and non-human primates (NHP) as amplifier hosts, with humans occasionally serving as unintentional hosts [2].

Despite the absence of urban cases due to the brief eradication of *Ae. aegypti* in Brazil until the 1960s, the virus remained endemic in forest areas of Northern and Midwestern Brazil (the Amazon Forest and Cerrado, respectively), and is occasionally responsible for epizootics and human cases of yellow fever (YF) [2,3].

The overgrowing human action in natural landscapes provokes an imbalance in ecosystem dynamics, exposing hosts, vectors, and known and unknown viruses to humans. In late 2016, the largest YF outbreak in Brazil in eight decades began in the countryside of Minas Gerais state and swiftly spread to other states of the Southeast Region, which is the most populated in the country and is within the Atlantic Forest biome. Until then, this Region, which registered 1865 human cases and 744 total deaths between 2016 and 2018, was not included under the Brazilian YFV vaccination program [4,5].

Since NHPs are used as sentinels in sylvatic YFV surveillance due to their high susceptibility to the virus, the occurrence of epizootics implies viral circulation. It warns of the urgency to strengthen prevention and control measures, such as improving vaccination coverage in the area. The late detection and response could end in a severe outbreak, as was recently registered in Brazilian Atlantic Forest [4,6,7,8].

Entomo-virological surveillance can also be used as an important tool for the early detection of viral circulation and to find an epidemiological link to epizootics and human cases in investigation. Whereas entomological collection is focused on main and potential vectors of YFV in a specific region, viral detection in mosquitoes helps to foresee the escalation of an outbreak and could define the possible vectors implicated in YFV transmission [7,8,9,10].

The possibility of urban YFV re-emergence in Brazil is feasible due to the YFV’s current dispersion over almost the entire country. In this scenario, sylvatic vectors are closer to urban areas, and opportunistic mosquitoes with vectorial potential could act as bridge vectors between the two transmission cycles [11,12], reinforcing the importance of implementing entomo-virological surveillance. Here, we present a retrospective study of YFV genomic investigation in 2904 *Aedes*, *Haemagogus*, and *Sabethes* genera mosquitoes, grouped into 246 pools and collected by the entomo-virological survey of the Brazilian Ministry of Health in six Brazilian states between 2016 and 2017.

## 2. Material and Methods

### 2.1. Mosquitoes Collection and Taxonomic Identification

The mosquito samples used in this study came from the YFV entomo-virological survey of the Brazilian Ministry of Health. They were collected in six states (Bahia, Goiás, Mato Grosso, Minas Gerais, Pará, and Tocantins) from four of the five Brazilian Regions between January 2016 and April 2017.

Mosquitoes were collected by human attraction using hand nets, a polyester net bag of 30 cm in diameter with a 30 cm aluminum handle commonly used by our entomological surveillance team. The number of collectors ranged from two to four persons, depending on the municipality. Collections were done in the morning, preferably during the hours of highest sylvatic YFV vector density, between 9 am and 4 pm, in wild outdoor environments.

The collected mosquitoes were transferred by oral suction to the identified cryotubes which were stored in liquid nitrogen at −196 °C and transported to the Department of Arbovirology and Hemorrhagic Fevers of the Evandro Chagas Institute prior to taxonomic identification and further analysis.

On a −20 °C refrigerated table and using a stereo microscope Stemi 2000-C (Zeiss, Oberkochen, Germany), mosquitoes were morphologically identified using dichotomous keys [13,14,15,16,17,18] to the species level and organized in pools with 1 to 30 specimens, based on species, date, and site of collection.

### 2.2. Mosquitoes Maceration

Based on an adapted protocol [19], each pool was eluted in 1 mL of a solution composed of 77 mL of 1X Dulbecco’s Phosphate Buffered Salino (Thermo Fisher Scientific, Waltham, MA, USA), 20 mL of Fetal Bovine Serum (Thermo Fisher Scientific), and 3 mL of an antibiotic solution of penicillin (100 U/mL), streptomycin (10 mg/mL) and fungizone (2.5 mg/mL). Then, a 3 mm tungsten bead was added to each pool, which was macerated using the TissueLyser II system (Qiagen, Hilden, Germany) for 2 min at 25 Hz.

### 2.3. RNA Extraction

Pools were centrifuged at 13,000× *g* for 10 min and 200 µL of supernatant was used for RNA extraction, which was performed with the Maxwell^®^ 16 Viral Total Nucleic Acid Purification Kit (Promega, Madison, WI, USA) in the Maxwell^®^ 16 System (Promega) instrument. Alternatively, the QIAamp viral RNA Kit (Qiagen) was used. Since these are mosquito samples, we used the *Escherichia coli* bacteriophage MS2 as a noncompetitive internal control RNA, which was added in a 2 µL volume in each sample.

### 2.4. Real-Time Reverse Transcription Polymerase Chain Reaction (RT-qPCR)

The assay was performed using the QuantiTect^®^ Probe RT-PCR (Qiagen) (Thermo Fisher Scientific) and specific primers and a probe for the YFV 5′ untranslated region [20]. The 25 µL reaction was composed of 12.5 µL of a 2X QuantiTect Probe RT-PCR Master Mix, 5.75 µL of nuclease-free water, 0.5 µL of 20 µM forward primer (YFallF, 5′-GCTAATTGAGGTGYATTGGTCTGC-3′), 0.5 µL of 20 µM reverse primer (YFallR, 5′-CTGCTAATCGCTCAAMGAACG-3′), 0.5 µL of 10 µM probe (YFallP, 5′-FAM-ATCGAGTTGCTAGGCAATAAACAC-TMR-3′), 0.25 µL of the 1X QuantiTect RT Mix enzyme, and 5 µL of extracted RNA.

For the noncompetitive internal control RNA detection, the 25 µL reaction had the same composition as the YFV assay, but with the following set of primers and probe: MS2 forward (5′-CATAAGTTAGATGGCCGTCTGT-3′, 50 µM), MS2 reverse (5′-TAGAGACGACAACCATGCCAAAC-3′, 50 µM), and MS2 probe (5′-VIC-TCCAGACAACGTGCAACATATCGCGACGTATCGTGATATGG -BHQ1-3′, 10 µM) [21].

In a 7500 Fast Real-Time PCR system (Thermo Fisher Scientific), the RT-qPCR assays were performed under the following cycling conditions: an initial RT step at 50 °C for 30 min, a denaturation step at 95 °C for 2 min, 45 cycles of 15 s at 95 °C and a final extension step of 1 min at 60 °C. Each sample was analyzed in duplicate and considered as positive when the average cycle threshold (Ct) value was less than 37 for both assays. The assay was validated by positive (YFV-infected mice brain tissue) and negative (nuclease-free water) controls.

### 2.5. Nucleotide Sequencing

In a joint initiative, the AR843690 sample, a pool of *Aedes albopictus* mosquitoes, was sequenced using the MinION^®^ sequencing device (Oxford Nanopore Technologies, Oxford, Oxfordshire, UK) by the Oswaldo Cruz Foundation (FIOCRUZ). The genome assembly was also performed by FIOCRUZ following the methodology described in Giovanetti et al. [22].

Other samples were prepared for sequencing by synthesizing first and second strands of complementary DNA, which were obtained with the cDNA Synthesis System Kit (Roche Diagnostics, Basel, Switzerland) and 400 µM Roche random primer. Agencourt AMPure XP Reagent Kit (Beckman Coulter, Brea, CA, USA) magnetic beads were used for cDNA purification and Nextera XT DNA Library Preparation Kit (Illumina, San Diego, CA, USA) for cDNA library preparation, with 1 ng of cDNA input. Quantification of cDNA was assessed using Qubit 2.0 Fluorometer (Thermo Fisher Scientific), and the fragments’ size range was evaluated using a 2100 Bioanalyzer Instrument (Agilent Technologies, Santa Clara, CA, USA). Sequencing was performed on the MiniSeq platform (Illumina) using the MiniSeq High Output Kit (300 cycles) based on 150 bp paired-end technology.

### 2.6. Bioinformatic Analysis

Genome assembly was carried out through a de novo methodology using IDBA-UD v.1.1.3 (k-mers 20, 40, 60, 80, and 100) [23] and SPAdes v.3.15.4 (k-mers 21, 33, 55, and 77) [24]. Contigs were merged using the SeqMan Pro tool in the Lasergene 11 Core Suit software [25], and then aligned against National Center for Biotechnology Information (NCBI) Reference Sequence (RefSeq) database Release 211 by DIAMOND v2.0.15 using blastx [26] with a 10^−3^ e-value threshold. The contigs were inspected with MEGAN6 [27] to identify those corresponding to YFV. Using Geneious v.9.1.8 software [28], contigs were inspected and mapped to reference (NC_002031), and then to raw data to increase coverage, both with Geneious Mapper. A multiple sequence alignment (MSA) of the complete YFV genome was performed using Mafft v.7 [29]. The five YFV genomes obtained were compared to 82 YFV sequences from arthropods, humans, and NHP.

Phylogenetic inference with maximum likelihood (ML) analysis with 1000 bootstrap iterations [30] was performed using GTR + F + R2 as a substitution model defined by IQ-TREE v.2 [31]. The resulting tree was rooted at the midpoint. Visualization was performed using FigTree v.1.4.4 [32] and Inkscape v.1.1 [33].

## 3. Results

### 3.1. Collection and Taxonomic Identification

**Figure 1:**
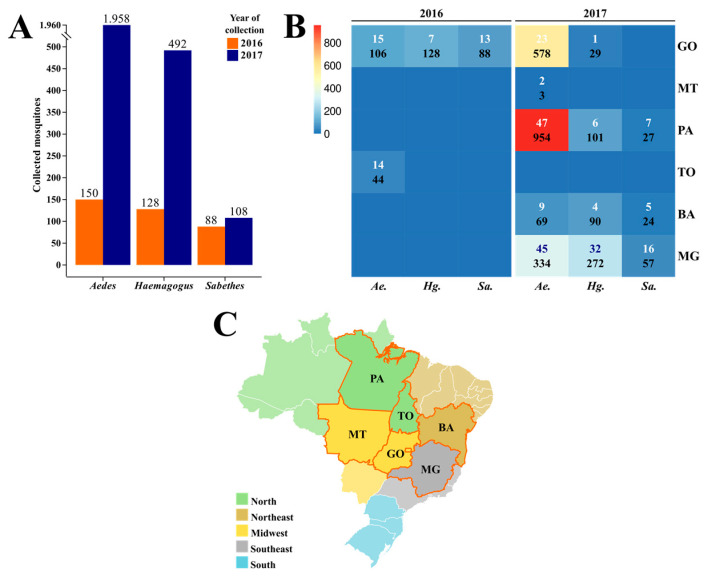
Total collected mosquitoes per year and their genera (**A**), distribution of total pools of mosquitoes (above) and total specimens (below) per genera and Brazilian state of collection (**B**), and map of location of the respective Brazilian states of collection (**C**). BA: Bahia; GO: Goiás; MT: Mato Grosso; MG: Minas Gerais; PA: Pará; and TO: Tocantins.

A total of 2904 mosquitoes of the *Aedes*, *Haemagogus*, and *Sabethes* genera were collected between January 2016 and April 2017 (Figure 1A) from six Brazilian states: Goiás and Mato Grosso (Midwest), Pará and Tocantins (North), Bahia (Northeast), and Minas Gerais (Southeast) (Figure 1B,C). They were grouped into 246 pools.

Mosquitoes of the *Aedes* genus were the most frequently collected in 2016 and 2017, representing 2088 specimens distributed in 155 pools, mostly from Goiás (38 pools/684 specimens) and Pará (47 pools/954 specimens). Although Minas Gerais was the state with more grouped pools (63 pools), Pará had the most specimens collected during this study, with samples dating only from 2017. On the other hand, Mato Grosso was the state with the fewest mosquitoes sampled, which were only from the *Aedes* genus. Goiás was the only state with mosquitoes sampled in both years.

Mosquitoes from the *Haemagogus* genus were organized into 50 pools with 620 specimens. Goiás was the only state from which *Haemagogus* mosquitoes were collected in 2016 (seven pools/128 specimens), and most of those collected in 2017 were from Minas Gerais (32 pools/272 specimens). From the *Sabethes* genus, 196 specimens were collected and grouped into 41 pools. Following a similar pattern as the *Haemagogus* genus, in 2016, *Sabethes* mosquitoes were collected only in Goiás (13 pools/88 specimens) and mostly in Minas Gerais in 2017 (16 pools/57 specimens). Goiás was the only state with no *Sabethes* mosquitoes collected in 2017. A description of each pool from 2016 and 2017 is available in Table S1 and Table S2, respectively.

### 3.2. RT-qPCR Detection

The 246 samples were tested for YFV and noncompetitive internal control RNA by RT-qPCR. The YFV amplicons were detected (positive) in 26 samples (Table 1). The MS2 were detected in all samples ranging from 16.95 to 28.55, validating our assays.

**Figure 2:**
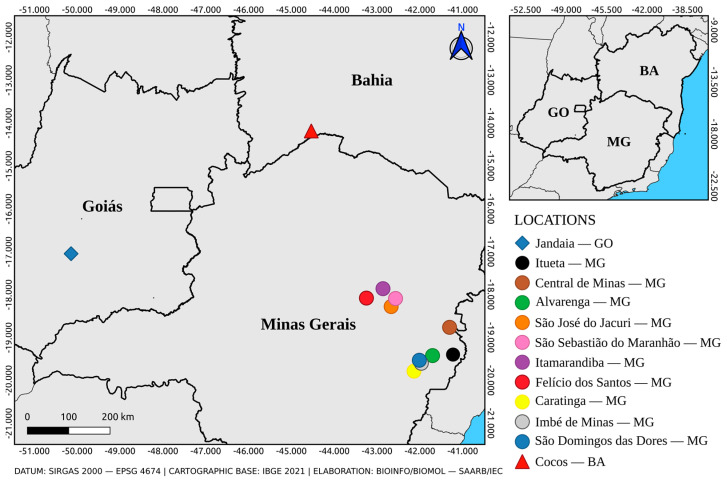
Municipalities of origin of the positive samples for YFV at RT-qPCR.

From the 26 positive pools, 5 had mosquitoes collected in 2016, all from Jandaia (Goiás—Cerrado), and were sampled over a two-day interval. The other 21 samples were from 2017, and only 1 was sampled in Bahia state (Cocos—Cerrado), while all the others were captured in 10 different municipalities from Minas Gerais state (Atlantic Forest), sampled over a 10-day interval (Figure 2).

The five positive samples from Jandaia consist of four pools of *Hg. janthinomys* and one *Sa. glaucodaemon*, and their Ct value in duplicate ranged from 18.19/18.35 to 31.05/31.49. The only positive pool from Cocos, a *Hg. janthinomys* pool, had specimens collected during three days in March 2017 and a Ct value in duplicate of 28.62/28.64.

Minas Gerais was the state with the most positive samples, with twenty pools collected in 2017, mainly from Alvarenga, with five positive pools, and Caratinga, and Ituêta, with three positive pools each. Their Ct value in duplicate ranged from 23.82/23.93 (pool of *Ae. albopictus* from Ituêta) to 35.46/35.75 (pool of *Ae. albopictus* from São Domingos das Dores). The 20 samples consisted of seven pools of *Hg. janthinomys*, five of *Ae. albopictus*, two of *Ae. argyrothorax*, two of *Ae. scapularis*, two of *Ae. serratus*, one of *Hg. leucocelaenus*, and one of *Aedes* sp. Between those samples, there were nine with only one to two specimens pooled, including two pools of *Ae. albopictus*, two of *Ae. argyrothorax*, and two of *Ae. serratus*.

### 3.3. Phylogenetic Analysis

Five of the 26 positive samples met the quality control standards and were submitted to whole genome sequencing. The quality control standards are influenced by viral load, which is not usually high in mosquito samples. Three sequences were from *Hg. janthinomys* from Jandaia (Goiás), one from *Hg. janthinomys* from Alvarenga (Minas Gerais), and one from *Ae. albopictus* from Ituêta (Minas Gerais). Genomes were deposited in GenBank under the following accession numbers: OQ932914 (AR831907), OQ932915 (AR831908), OQ932916 (AR831909), MH329655 (AR843690), and MF370530 (AR843721).

**Figure 3:**
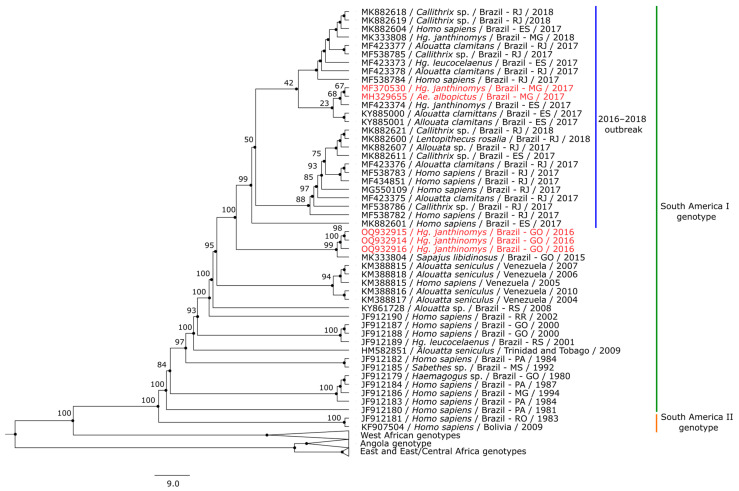
Maximum likelihood phylogenetic inference using the GTR + F + R2 nucleotide substitution model based on the YFV complete genome of 87 sequences, including five obtained from mosquitoes in our study (in red). African genotype clades are collapsed. Each record includes the sequence accession number, host species, country (and state for Brazilian sequences), and collection year. GO: Goiás; ES: Espírito Santo; MG: Minas Gerais; MS: Mato Grosso do Sul; PA: Pará; RJ: Rio de Janeiro; RO: Rondônia; RR: Roraima; RS: Rio Grande do Sul.

The five new genome sequences belonged to the South America I clade. The two sequences from Minas Gerais were close to other sequences from the recent Brazilian outbreak and the three sequences from Goiás grouped together with another viral strain from the state of Goiás, collected in 2015 (Figure 3). Information about sequences included in this study are available in Table S3.

## 4. Discussion

In the last eight decades, YFV has been only maintained in its sylvatic cycle in Brazil and occasionally re-emerged in North, Midwest, and Southern Regions, indicating its constant tendency to spread to the Southeast, which was observed in the recent 2016–2018 outbreak [2,3,4,5,34,35,36,37].

In the Americas, forest-living mosquitoes of the *Haemagogus* and *Sabethes* genera are considered the primary and secondary vectors of sylvatic YFV, respectively, and have a wide geographical distribution in Brazil [2,8,10]. In our study, *Haemagogus* mosquitoes represent 21.35% of the 2904 collected mosquitoes and 20.3% of the 246 pools. On the other hand, mosquitoes of the *Sabethes* genus represent 6.75% of the 2904 collected mosquitoes and 16.6% of the 246 pools. Most mosquitoes of these genera are from Goiás and Minas Gerais, and were collected in 2016 and 2017, respectively. In addition, half of the 26 positive samples are from mosquitoes of the *Haemagogus* genus—and from these, only one is *Hg. leucocelaenus*—and another one is from the *Sabethes* genus, a pool of *Sa. glaucodaemon* from Jandaia. 

In Brazil, the *Hg. janthinomys* species has been identified as the primary vector of sylvatic YFV [2,8,10]. There are descriptions of natural infection of this species with YFV during late-twentieth-century Brazilian outbreaks [38,39]. *Hg. leucocelaenus*, a common secondary vector defined as a primary vector in forests in Southern Brazil in the absence of *Hg. janthinomys* [34,35], was implicated as the main vectors in the recent outbreak in the Southeast Region [10]. Our findings from Midwest Brazil (Cerrado biome) stress the potential for active transmission in the Region, which is already considered an endemic area for YFV and has the presence of main vectors of the virus. On the other hand, the *Haemagogus*-positive pools from the Atlantic Forest support the vector presence and its participation in the maintenance of the 2016–2018 outbreak [10].

The positive *Hg. janthinomys* pool from Cocos, in the Bahia Cerrado, on the border with Northwestern Minas Gerais, corroborates the YFV spread from Minas Gerais to Bahia and the feasibility of YFV cases occurring in the region, which has a low vaccination coverage in humans, and the presence of main vectors and susceptible NHP [22].

Mosquitoes of the *Sabethes* genus in general are considered secondary vectors of YFV and could present a potential role in the transmission of YFV in the absence or in low density of primary vectors. However, studies on the detection of YFV in *Sa. glaucodaemon* species and its role in the sylvatic YFV transmission cycle are scarce [2,8]. Due to the detection of YFV in *Hg. janthinomys* from the same municipality, it is unlikely that *Sa. glaucodaemon* participates in the active transmission of the virus there.

*Aedes* mosquitoes represent the majority of samples analyzed in this study: they comprised 71.9% of the 2904 collected mosquitoes and 63% of the 246 pools. This genus comprises species of great importance in public health, such as *Ae. aegypti* and *Ae. albopictus*. *Ae. aegypti* was the first vector discovered to be related to a virus (YFV) [40,41], representing a revolution in the understanding of some viruses now known as arboviruses, and was related to the urban transmission cycle of YFV in Brazil until 1942, when the last urban YFV outbreak occurred in the country [2]. Since the late 1980s, *Ae. albopictus* has been detected and has spread in Brazil, and today more than 1000 municipalities have reported its presence in the peridomicile and adjacent natural or modified environments. Coincidentally, the highest infestation indices for *Ae. albopictus* in Brazil are reported mainly in the Southeast Region, where the outbreak occurred [42]. These species are very opportunistic and strongly anthropophilic species, able to colonize a wide range of habitats, and is adapted to tropical and temperate regions of the world [11,43].

The YFV was previously described in two of the positive *Aedes* species from the Atlantic Forest: *Ae. scapularis* [2,10,44], and *Ae. serratus* [35]. Indeed, this is the first YFV detection in *Ae. argyrothorax*; however, the real importance of YFV spillover in these three species in maintaining YFV in nature remains to be determined. Entomo-virological surveillance and experimental studies on vectorial competence could certainly contribute to enlightening their role in sylvatic YFV maintenance. 

Based on experimental studies, *Ae. albopictus* has been incriminated as a potential vector for YFV transmission [11,43,45]. Still, until 2018, when the Evandro Chagas Institute publicly announced these first detections [46], there was no scientific evidence of natural infection by YFV in this mosquito species.

For a mosquito species to be considered as a potential vector for arbovirus transmission, it is necessary to combine criteria of all of the physiological and ecological factors of vector, host, pathogen, and environment that determine the vector status of a given arthropod population. The criteria are: (a) isolation of a specific virus from specimens collected in nature; (b) demonstration of infection in the mosquito following experimental feeding on a viremic host or virus suspension; (c) demonstration of transmission of virus by bite to a vertebrate host or demonstration of transmission through excretion of the virus in salivary fluids; and (d) field evidence confirming association of the mosquito species with the vertebrate population in which the virus infection is occurring [47,48]. Even with a considerable number of positive *Ae. albopictus* samples among the Minas Gerais pools, no subsequent detection of naturally infected *Ae. albopictus* in the region was made, thus the species was not implicated as a vector involved in the recent Brazilian YFV outbreak, during which reported cases were associated with the sylvatic transmission cycle [10].

Although it was not related to the outbreak, the vector competence of *Ae. albopictus* was experimentally confirmed, and its biological characteristics, including the easy movement between both sylvatic and peri-urban environments associated with cases in humans and NHP, and its geographic distribution in Brazil, reinforce the concern about the risk of YFV re-urbanization in the country [11,43,45].

The natural YFV infection detected in *Ae. albopictus* pools in this study suggests that this mosquito species could play the role of a bridge vector linking the sylvatic YFV to the urban cycle and establishing an intermediary transmission cycle, as documented in Africa with *Ae. simpsoni* and other *Aedes* mosquito species [3,12], once the low vector capacity could be overcome by other factors such as high vector density, high human-biting rate, and high daily survival rates [49].

In the phylogenetic analysis, the five sequences obtained in this study are clade-related to others from the 2016–2018 outbreak within South America I genotype of YFV [5,50,51,52]. The three Midwest sequences, previous to the outbreak, are related to a 2015 sequence obtained from an NHP sample collected in Novo Brasil, a municipality from Goiás, located 175 km away from Jandaia. These sequences from Goiás clustered in a sister clade to the outbreak clade, confirming the already described topology of previous phylogeny positioning the NHP as related to the recent Southeast sequences [51], and could reinforce the possibility of spread from the Midwest to the Southeast [22].

The other two sequences are from geographically close Minas Gerais municipalities and clustered within the outbreak clade, close to a sequence which was obtained from a *Hg. janthinomys* and two other sequences obtained from NHP, all in 2017 from the Espírito Santo state. The proximity of Minas Gerais municipalities to Espírito Santo could explain their closeness due to virus spread, even with the multiple viral exchanges observed through the deposited sequences during the outbreak, which actually justifies the existence of several sub-clades with the 2017–2018 sequences in the phylogenetic analysis [52].

## 5. Conclusions

Entomo-virological surveillance has proven to be a crucial strategy in YFV surveillance in Brazil, linking and confirming data of the virus’ active transmission to NHP and humans, as well as shedding light on the involvement of unusual or potential vectors in the maintenance of YFV, which may pave the way for new studies. 

Analysis of main and potential vectors helps to understand their participation in the spread and maintenance of sylvatic YFV and the possibility of an oncoming re-emergence of urban YFV in Brazil. Furthermore, it could highlight the urgency to strengthen the monitoring of syndromic surveillance and NHP deaths, vaccination coverage, and vector control measures, including *Ae. aegypti* and *Ae. albopictus*.

## Figures and Tables

**Table 1:** Samples with YFV amplicon detection at RT-qPCR.

Sample ID	Species	Specimens Per Pool	Location	Collection Date	RT-qPCR Ct Value
AR831906	*Hg. janthinomys*	30	Jandaia—GO	24-Sep.-2016	26.02/26.31
AR831907	*Hg. janthinomys*	30		24-Sep.-2016	23.05/23.98
AR831908	*Hg. janthinomys*	28		24-Sep.-2016	20.99/21.53
AR831909	*Hg. janthinomys*	7		26-Sep.-2016	18.19/18.35
AR831914	*Sa. glaucodaemon*	30		24-Sep.-2016	31.05/31.49
AR843690	*Ae. albopictus*	25	Ituêta—MG	14-Jan.-2017	23.82/23.93
AR843692	*Ae. scapularis*	25			31.08/31.08
AR843693	*Ae. argyrothorax*	2			32.83/34.74
AR843713	*Hg. janthinomys*	15	Central de Minas—MG	13-Jan.-2017	32.07/32.40
AR843715	*Ae. albopictus* ♀	4	Alvarenga—MG	15-Jan.-2017	33.49/35.06
AR843716	*Ae. argyrothorax*	2			32.44/33.31
AR843717	*Aedes* sp.	1			35.14/35.95
AR843720	*Hg. janthinomys*	25			28.95/29.20
AR843721	*Hg. janthinomys*	25			28.33/28.55
AR843728	*Hg. janthinomys*	19	São José do Jacuri—MG	16-Jan.-2017	31.86/32.32
AR843738	*Ae. scapularis*	3	São Sebastião do Maranhão—MG	17-Jan.-2017	35.03/35.12
AR843741	*Hg. leucocelaenus*	2			24.07/24.10
AR843745	*Ae. serratus*	1	Itamarandiba—MG	18-Jan.-2017	35.11/35.80
AR843765	*Hg. janthinomys*	6	Felício dos Santos—MG	20-Jan.-2017	32.43/32.59
AR843771	*Ae. albopictus* ♀	2	Caratinga—MG	10-Jan.-2017	34.96/35.28
AR843772	*Ae. serratus*	2			31.78/32.54
AR843777	*Hg. janthinomys*	1			27.60/27.60
AR843807	*Hg. janthinomys*	4	Imbé de Minas—MG	16-Jan.-2017	34.52/35.09
AR843821	*Ae. albopictus* ♀	1	São Domingos das Dores—MG	19-Jan.-2017	35.46/35.75
AR843829	*Ae. albopictus* ♀	4			35.12/35.25
AR845803	*Hg. janthinomys*	18	Cocos—BA	17,18,20-Mar.-2017	28.62/28.64

♀: female; *Ae.*: *Aedes*; *Hg*.: *Haemagogus*; *Sa*.: *Sabethes*; sp.: species not defined; BA: Bahia; GO: Goiás; MG: Minas Gerais; Ct: cycle threshold value.

## Data Availability

The five sequences were deposited in GenBank under the following accession numbers: OQ932914 (AR831907), OQ932915 (AR831908), OQ932916 (AR831909), MH329655 (AR843690), and MF370530 (AR843721).

## Supplementary Materials

The following supporting information can be downloaded at: https://www.mdpi.com/article/10.3390/tropicalmed8060329/s1, Table S1: Description of pools of mosquitoes from 2016; Table S2: Description of pools of mosquitoes from 2017; Table S3: Sequences used in the phylogenetic analysis.
